# Editorial: Novel therapeutic targets in autoimmune diseases: intestinal microbiota and adaptive immunity regulation

**DOI:** 10.3389/fimmu.2026.1779062

**Published:** 2026-01-20

**Authors:** Caio César de Souza Alves, Sandra Bertelli Ribeiro de Castro, Alessa Sin Singer Brugiolo

**Affiliations:** 1 Faculdade de Medicina, Universidade Federal dos Vales do Jequitinhonha e Mucuri, Teófilo Otoni, Brazil; 2 Departamento de Fundamentos, Métodos e Recursos em Fisioterapia, Faculdade de Fisioterapia, Universidade Federal de Juiz de Fora, Juiz de Fora, Brazil

**Keywords:** acupuncture, autoimmune diseases, dysbiosis, gastrointestinal microbiome, immunologic factors, short-chain fatty acids, therapeutic targets

Autoimmune diseases comprise more than 80 different diseases and affect an increasing number of people, with women between the ages of 20 and 40 being the most affected. Autoimmune diseases are a significant burden on the healthcare system and are characterized by the presence of autoreactive T lymphocytes or autoantibodies, which may be physiologically or pathologically induced. It is known that genetic and environmental factors influence the onset of immune response in autoimmune diseases, although the etiology is poorly understood.

The papers of this Frontiers in Immunology Research Topic contribute to the expansion of knowledge regarding interactions occurring between the immune system and the intestinal system, providing insights into possible pathogenesis mechanisms and new therapeutic possibilities for autoimmune diseases.

It is already known that controlling adaptive immunity results in better disease outcomes, and Bredberg et al. reasoned that understanding must expand for the vast complexity of autoimmune diseases. The authors highlight the interaction between the gut microbiota, adaptive immunity, and the central nervous system as a key factor in the pathogenesis of these diseases, underling that the intestinal barrier serves as a sophisticated defense regulator. In that regard, future therapies should consider the complex network of interactions, including DNA repair, the intestinal microbiota, and the central nervous system.

Li et al. present a review article on the crucial link between metabolites derived from the gut microbiota and the modulation of the Treg/Th17 balance. The imbalance between regulatory T cells (Treg), which promote immune tolerance, and helper T cells 17 (Th17), which drive inflammation, is a central mechanism in the pathogenesis of autoimmune conditions. The authors show clinical and preclinical evidence demonstrating how microbial metabolites, such as short-chain fatty acids (SCFA), secondary bile acids, tryptophan, and other aromatic metabolites, regulate this cellular dynamic in Rheumatoid Arthritis, systemic lupus erythematosus, Graves’ disease, autoimmune hepatitis, and myasthenia gravis. Furthermore, the research evaluated microbiome-targeted interventions, including probiotics, diet, and fecal microbiota transplantation, in autoimmune diseases.

Kou et al. explored, in particular, how the gut microenvironment plays a fundamental pathophysiological role in driving Inflammatory Bowel Disease, a multifaceted, chronic inflammatory condition with its underlying pathophysiological mechanisms not yet fully elucidated. The disturbance of beneficial microbiota and the proliferation of pathogenic organisms initiate a cascade that compromises intestinal homeostasis. Reduced protective metabolites and impaired tight junction integrity facilitate microbial translocation and sustained immune responses, perpetuating chronic inflammation and affecting the gastrointestinal tract through complex interactions. Additionally, the paper discusses the limitations of current therapeutic strategies, which focus on symptomatic relief, and proposes an integrated approach to restore intestinal homeostasis and provide long-term disease control.

Zhao et al. investigated the theoretical foundation for further elucidating the pathogenesis of Rheumatoid Arthritis using the collagen-induced arthritis (CIA) mouse model. The authors observed that CIA mice exhibited significant dysregulation of the microbiota, characterized by a reduction in butyrate-producing bacteria (such as the Lachnospiraceae_NK4A136 and Roseburia group) and lower levels of short-chain fatty acids (SCFA). Additionally, a significant increase in CD8^+^ T cells in local intestinal tissues, such as mesenteric lymph nodes, correlated with decreased SCFA and intestinal dysbiosis, resulting in an imbalance of T/B cells in the spleen and ultimately leading to the occurrence, development, and accelerated arthritis progression. The authors suggested that the intestinal microbiota–SCFA–CD8^+^ T cell axis may be fundamental in the development and worsening of arthritis, focusing on restoring the balance of gut microbiota and regulating the functions of CD8^+^ T cells and B cells, providing new ideas for the future treatment of Rheumatoid Arthritis.

Finally, Li et al. investigate the mechanism by which acupuncture, a traditional Chinese therapy, attenuates experimental autoimmune thyroiditis in rats. The study demonstrates that acupuncture improves thyroid dysfunction and reduces apoptosis of follicular epithelial cells by modulating the intestinal microbiota-palmitoleic acid metabolism axis. Specifically, acupuncture intervention reverses microbial dysbiosis and metabolic disorders by increasing the abundance of Prevotella bacteria and reducing serum palmitoleic acid levels, suggesting that these may be key therapeutic targets for treatment of autoimmune thyroiditis.

While the knowledge of disease mechanisms has incredibly advanced, current therapy for autoimmune diseases still shows few advances, and most patients cannot be cured. Conventional pharmacotherapy, surgical intervention, and adjunctive therapies present limitations and, in general, involve controlling inflammation or targeting a single pathway. Therefore, developing new therapeutic approaches should consider not only the complex network of interactions but also multidimensional interventions, systemic modulation strategies, and the restoration of intestinal homeostasis.

In this sense, recent research has explored not only the therapeutic potential of modulating the immune system but also the modulation of the gut microbiota and the central nervous system. New therapeutic possibilities include microbiome remodeling, metabolite supplementation, immune microenvironment reconstruction, and barrier repair, as well as emerging technologies such as multi-omics, gene editing, and nanoparticle delivery systems.

Despite this innovative approach, the safety and ethical aspects of treatments must be addressed, as they exhibit variability in effectiveness across individuals and may carry potential risks. Moreover, the challenge of developing treatments that present better results, with fewer side effects and lower costs, remains a great hope for improving the prognosis of autoimmune diseases.

